# Morpho-cultural and molecular characterization of *trichoderma* species from the northwestern himalayan apple rhizosphere of India

**DOI:** 10.1038/s41598-025-12086-4

**Published:** 2025-07-20

**Authors:** Sana Surma, Sumaira H., Misbah M., M. S. Dar, Bilal A. Padder, Imran Khan, Khalid Mushtaq, Maheen M., Sehla K., Asha Nabi, Mushtaq A. Lone, Snober S. Mir, Ozer Callis, Mehraj D. Shah

**Affiliations:** 1 Plant Virology and Molecular Pathology Lab, Division of Plant Pathology, Sher-e-Kashmir, University of Agricultural Sciences and Technology of Kashmir, Shalimar, Srinagar, 190025 Jammu and Kashmir India; 2 Department of Biosciences and Bioengineering, Integral University, Lucknow, UP India; 3 Division of Statistics, Sher-e-Kashmir University of Agricultural Sciences and Technology of Kashmir, Shalimar, Srinagar, 190025 Jammu and Kashmir India; 4 Division of Fruit Science, Sher-e-Kashmir University of Agricultural Sciences and Technology of Kashmir, Shalimar, Srinagar, 190025 Jammu and Kashmir India; 5 Department of Plant Protection, Akdeniz University, Antalya, Turkey; 6 Division of Plant Pathology, Faculty of Horticulture, Sher-e-Kashmir, University of Agricultural Sciences and Technology of Kashmir, Shalimar, Srinagar, 190025 Jammu and Kashmir India

**Keywords:** *Trichodema* species, Morphological characterization, Cultural characterization, Internal transcribed spacer (ITS), translation elongation factor 1-α (*TEF1-α*), RNA polymerase B subunit II (*RPB2*), Biological techniques, Biotechnology, Evolution, Plant sciences

## Abstract

Plant disease management based on pesticide use has numerous detrimental effects on health and the environment. As a result, the adoption of environment-friendly disease management options is the best alternative to pesticide use. Therefore, the identification of locally available bio-agents like *Trichoderma* species using morpho-cultural and molecular approaches specifically targeting the internal transcribed spacer (ITS) region, translation elongation factor 1-alpha (*TEF 1-α*) and RNA polymerase B subunit II (*RPB2*) is necessary. In this study, we characterized 24 *Trichoderma* strains isolated from the apple rhizosphere. Significant variations were observed in the morpho-cultural characteristics of *Trichoderma* isolates and categorized them into four groups (I-IV) that were identified as *T. harzianum* complex, *T. koningiopsis*,* T. viride*, and *T. hamatum*, comprising 4, 4, 6 and 10 isolates, respectively. The concatenated sequence data set derived from the ITS region, *TEF 1-α* and *RPB2* grouped 24 *Trichoderma* isolates into 03 independent clades. Specifically, the sequencing based on ITS region grouped them into four sub-clades, which were identified as *T. harzianum* complex, *T. viride*,* T. asperelloides*, and *T. koningiopsis*, comprising 4, 6, 5 and 7 isolates, respectively, and two independent lineages, each represented by a single isolate. In contrast, sequencing of the *TEF 1-α* and *RPB2* genes grouped 24 *Trichoderma* isolates into two distinct clades and six sub-clades that were identified as *T. asperelloides*,* T. asperellum*,* T. hamatum*,* T. viride*,* T. koningiopsis* and *T. harzianum* complex, comprising 5, 5, 3, 4, 3 and 4 isolates, respectively. Thus, the final identification of 24 *Trichoderma* strains was achieved through a combined morpho-cultural and molecular approach, resulting in the identification of six species viz.,* T. koningiopsis*, *T. viride*, *T. asperellum*,* T. asperelloides*,* T. hamatum* and *T. harzianum* complex comprising 5, 5, 3, 4, 3 and 4 isolates, respectively in accordance with the reference sequences retrieved from NCBI. Notably, to our knowledge, this is the first report of *T. koningiopsis*, *T. viride*, *T. asperellum*,* T. asperelloides*, and *T. hamatum* from the apple rhizosphere.

**Supplementary Information:**

The online version contains supplementary material available at 10.1038/s41598-025-12086-4.

## Introduction

Apple (*Malus x domestica* L.) is a premier table fruit cultivated in the temperate regions of the world as well as in the cool highlands of sub-tropical regions such as East Africa and the Northeastern parts of India. In India, apple cultivation accounts for 55% of the total area and 75% of the total production under temperate fruits^1^. In India, the commercial cultivation of apples is primarily confined to Jammu and Kashmir, Himachal Pradesh, and some selected parts of Uttarakhand, Arunachal Pradesh, Manipur, and Sikkim. In Jammu and Kashmir, apple is the most significant fruit crop, covering an area of 16,47,42,000 ha with an annual production of 18,82,319 metric tons^2^. However, apple is prone to various fungal, bacterial, and viral diseases affecting its quality and production. Among these, fungal diseases inflict substantial losses on the crop. Notably, soil-borne diseases such as white root rot and collar rot caused by *Dematophora necatrix* and *Phytopthora cactorum*, respectively, are significant threats after scab to the apple industry globally, including in Jammu and Kashmir^3^.

Strategies like pesticides and biological control agents are being used to manage plant diseases, especially soil-borne diseases^4^. However, the excessive use of pesticides leads to contamination of soil and water as many components of pesticides are recalcitrant and persist in the environment for a longer duration^5^. As a result, the application of biological control agents for plant disease management is gaining popularity as a way to reduce or eliminate the use of synthetic pesticides^4^. Among the various biological control agents of fungal origin, the *Trichoderma* species is the most intensively studied fungal biocontrol agent. Its biocontrol activity is of immense importance to agriculture and the environment, without any harmful effects on plants, animals, and humans^6,7^.

The genus *Trichoderma* is classified under the subdivision: Pezizomycotina (Ascomycota); class: Sordariomycetes; family: Hypocreaceae^8,9^. Over the past 35 years, the number of recognized *Trichoderma* aggregate and phylogenetic species has increased from nine to approximately eighty, respectively, indicating its discovery phase. It shows that several new *Trichoderma* species are likely to be identified through the exploration of new niches and new geographical regions. The identification and phylogenetic classification of strains have been supported by the development of new molecular tools, making DNA sequence data essential for the accurate identification of species within *Trichoderma* or any other economically important genera^10,11^. Some of the *Trichoderma* strains like *T. viride*, *T*. *harzianum*, *T. longibrachiatum*, *T. hamatum*, and *T. koningii* are recognized for their efficient biocontrol ability to inhibit the growth of soil-borne plant pathogens, hence improving overall plant health^12,13^. These strains are opportunistic, avirulent plant symbionts and parasites of other plant pathogenic fungi, commonly found in soil and root ecosystems^14^. For instance, *Trichoderma virens* isolate Tvr4 and *Trichoderma harzianum* isolate Thr15 have shown high effectiveness against the mycelial growth and microsclerotial suppression of *Macrophomina phaseolina*, causing charcoal rot of strawberry^15^. Rifai made an initial attempt to understand the diversity in *Trichoderma* spp. by introducing the concept of 9 “species groups”^16^. As is usually the case with other fungal genera, species of *Trichoderma* were also defined originally based on morphology by Rifai and Bissett^16–18^. The cultural sporulation pattern varied considerably within and between the two species of *Trichoderma*. Although conidial shape and arrangement, and hyphal branching pattern helped in distinguishing species from each other, they failed to designate *Trichoderma* species. Seaby^19^ also reported the difficulties in differentiation of *Trichoderma* spp. using classical microscopic features alone since cultural morphology and spore size significantly varied on different media and incubation temperature, respectively. Moreover, variation among the isolates based on phialide size, and their arrangements is very less. However, the sporulation pattern and spore size within the species are highly variable. Consequently, the morphological data can lead to misidentification, with 30–50% of identified characteristics potentially erroneous^10^. To overcome these limitations, the research over the past many years has increasingly focused on application of molecular approaches, resulting in the re-classification of several species and strains^20^. Molecular methods particularly based on multiple gene sequencing enhance the accuracy of the fungal species identification. Internal transcribed spacer (ITS) region of ribosomal DNA (rDNA) is one of the most consistently used targets to identify a *Trichoderma* strain at the species level^21,22^but some of the meticulously related species of *Trichoderma* share the sequences of their ITS regions, therefore cannot differentiate all the *Trichoderma* species [International Sub-commission on *Trichoderma* and *Hypocrea* Taxonomy (http://www.isth.info)]^11,23^. Conversely, translation elongation factor 1-alpha (*TEF 1-α*) and RNA polymerase B II (*RPB2*) genes are more variable and can effectively reflect the differences within and among the groups of closely related species in *Trichoderma*. Consequently, a combination of multigene sequencing based on *TEF 1-α*, and *RPB2* genes, and the ITS region, proves invaluable for the proper identification of *Trichoderma* at the species level^11,23^. Besides, ITS, *TEF 1-α*, and *RPB2* sequencing identified *Trichoderma guizhouense* in Türkiye^24^.

Utilizing combinatorial strategies such as morpho-cultural and molecular studies based on DNA barcodes significantly enhances the capacity to accurately delineate various *Trichoderma* species^22,25^. Furthermore, as the identification of a plant disease/ pathogen is crucial for its effective management, the species identification of a biocontrol agent is equally essential for the development of commercial products. Therefore, keeping in view the lack of comprehensive studies regarding the variability of *Trichoderma* species in India particularly in Jammu and Kashmir, the present study aims to characterize the *Trichoderma* isolates collected from apple rhizosphere using conventional (morpho-cultural characteristics) and molecular methods based on ITS region, and *TEF 1-α* and *RPB2* gene sequencing.

## Results and discussion

### Morpho-cultural characterization of the *trichoderma* isolates

#### Colony characteristics

**Fig. 1:**
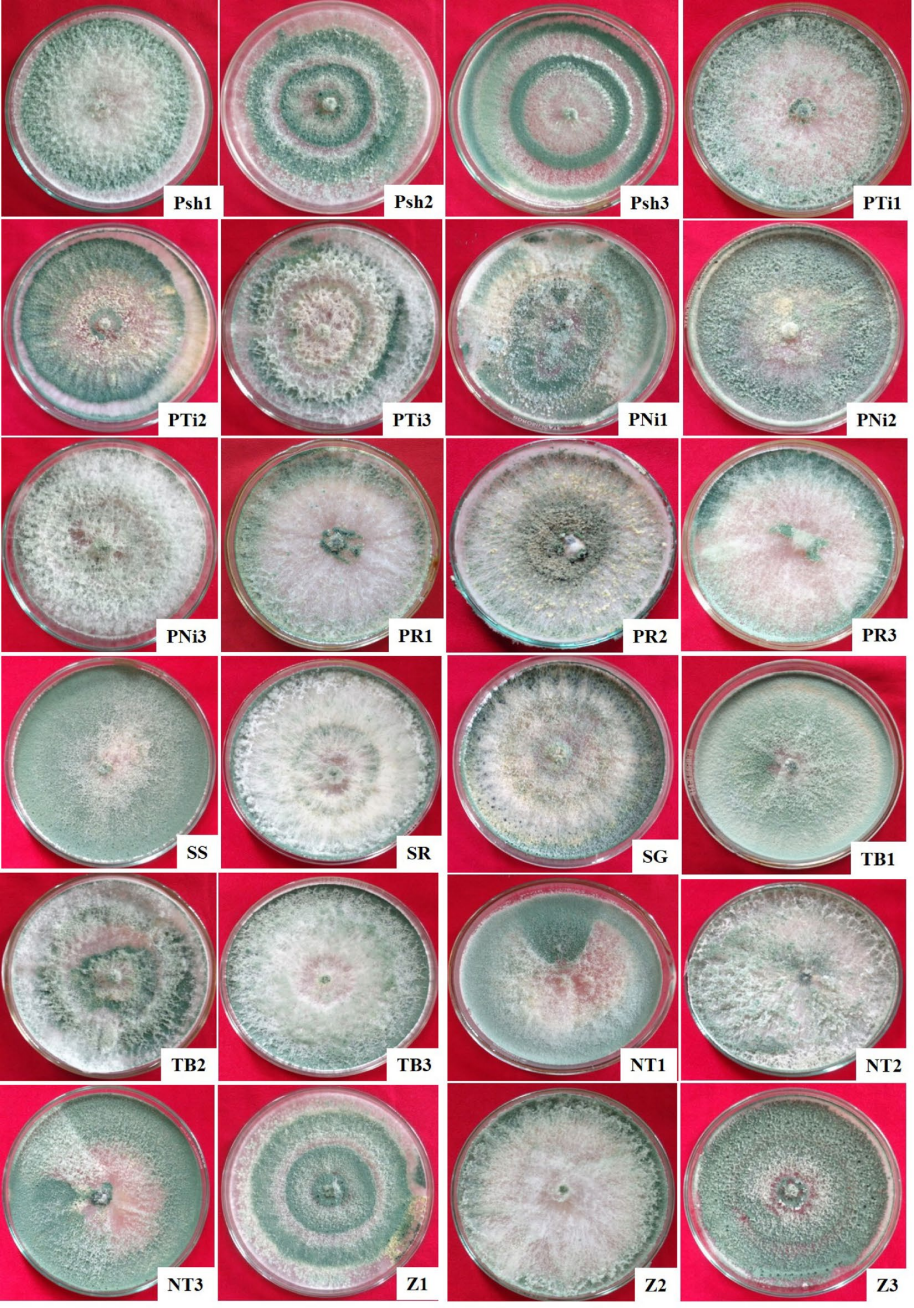
Mycelial growth of different *Trichoderma* isolates on potato dextrose agar medium after 5 days of incubation at 28 ± 1^o^C.

Different *Trichoderma* isolates cultured on potato dextrose agar (PDA) medium (HiMedia Lab. Pvt. Ltd., Mumbai, India) showed notable variations in colony characteristics viz., colony texture, margins, colour, and shape (Table 1; Fig. 1). Based on texture, *Trichoderma* isolates were divided into Group I (cottony), Group II (Fluffy), and Group III (Velvety) comprising of 33.33% of isolates (Psh2, Psh3, PTi1, PTi2, PR3, NT2, Z1, and Z2), 41.67% (Psh1, PTi3, PNi1, PNi3, PR1, PR2, SR, SG, TB2, and TB3) and 25.0% of isolates (PNi2, SS, TB1, NT1, NT3, and Z3), respectively (Table 1). Further categorization of the isolates based on colony margins yielded two groups: Group I (regular margins) and Group II (irregular margins) (Table 1). Notably, the majority of isolates (87.50%) namely Psh1, Psh2, Psh3, PTi1, PTi2, PNi2, PNi3, PR1, PR2, PR3, SS, SR, SG, TB1, TB3, NT1, NT2, NT3, Z1, Z2 and Z3 were accommodated in Group I, whereas Group II comprised of only three isolates viz., PNi1, PTi3, and TB2 accounting for 12.50% of the remaining isolates. Furthermore, *Trichoderma* isolates were categorized based on colony colour, into four groups: Group I (light green with white centre), Group II (dark green with white centre), Group III (Blackish green with white centre), and Group IV (green with dull white centre) accommodating 20.83% of isolates (Psh1, PTi1, PNi2, PR3 and TB1), 45.83% (Psh2, Psh3, PTi2, PTi3, PNi1, Z1, SS, TB2, NT1, NT3 and Z3), 16.67% (PR1, PR2, SG and Z2) and 16.67% (PNi3, SR, TB3 and NT2) of isolates, respectively (Table 1). Based on colony shape, the isolates were divided into four groups accommodating 4 (16.67%), 4 (16.67%), 6 (25.00%), and 10 (41.66%) isolates in Group I (Psh2, Psh3, PTi2 and Z1), Group II (PTi1, PR3, NT2 and Z2), Group III (PNi2, SS, TB1, NT1, NT3 and Z3), and Group IV (Psh1, PTi3, PNi1, PNi3, PR1, PR2, SR, SG, TB2 and TB3), respectively (Table 1). Present results were in close agreement with the observations of Mohiddin et al., Kumar et al., Meena et al. and Shah et al. around the world^26–29^ who noted similar textures among the colonies of various *Trichoderma* isolates including flat cottony, fluffy or raised and flat granular forms with either regular or irregular margins on PDA medium. Additionally it has been documented that the majority of *Trichoderma* isolates showed diverse colony colours like dark green (12 isolates), whitish green (9 isolates), light green (6 isolates), and yellowish green (3 isolates)^30^. It has also been observed that the colony colour of 10 selected isolates of *Trichoderma* varied from dark green to light green or black green or whitish green. Similarly, colour variation from dark green or light green to yellowish green with flat to raised growth patterns has also been reported by Kamaruzzaman et al.^31^. These observations were in close agreement with our results. Furthermore, the colony shapes exhibited notable diversity among different *Trichoderma* isolates. The production of green spore mass was observed at the centre of the Petri plates containing PDA medium, often forming 1–2 concentric rings or spreading across the plate, accompanied by yellowish or whitish aggregated spore mass or white mycelium with green conidia towards the edges. Some isolates formed white mycelium with limited green conidial formation in concentric rings near the centre or dispersed along the margin. These observations regarding the colony shape are in close agreement with the findings reported by Ghutukade et al.^32^.

**Table 1:** Grouping of *Trichoderma* isolates based on their colony characteristics.

Group	Colony characteristics^$^			
	Texture	Margins	Colour	Shape
I	**Cottony** (33.33%) Psh2, Psh3, PTi1, PTi2, PR3, NT2, Z1, Z2	**Regular** (87.50%) Psh1, Psh2, Psh3, PTi1, PTi2, PNi2, PNi3, PR1, PR2, PR3, SS, SR, SG, TB1, TB3, NT1, NT2, NT3, Z1, Z2, Z3	White centre with light green outer region (20.83%) Psh1, PTi1, PNi2, PR3, TB1	Green conidial production denser at centre than towards the margins in 1–2 concentric rings (16.67%) Psh2, Psh3, PTi2, Z1
II	**Fluffy** (41.67%) Psh1, PTi3, PNi1, PNi3, PR1, PR2, SR, SG TB2, TB3	**Irregular** (12.50%) PNi1, PTi3, TB2	White centre with dark green outer region (45.83%) Psh2, Psh3, PTi2, PTi3, PNi1, Z1, SS, TB2, NT1, NT3, Z3	White mycelia at the centre with less green conidial production dispersed near the margins (16.67%) PTi1, PR3, NT2, Z2
III	**Velvety** (25%) PNi2, SS, TB1, NT1, NT3, Z3		White centre with black green outer region (16.67%) PR1, PR2, SG, Z2	Green conidia distributed throughout the plate with yellow pigmentation or whitish aggregate of conidia around the centre (25.00%) PNi2, SS, TB1, NT1, NT3, Z3
IV			Dull white centre with green outer region (16.67%) PNi3, SR, TB3, NT2	White mycelia with limited green conidial formation in concentric rings near the centre or dispersed along the margins (41.67%) Psh1, PTi3, PNi1, PNi3, PR1, PR2, SR, SG, TB2 and TB3

^**$**^P- Pulwama, sh- Shadimarg, Ti- Tiken, Ni- Nikas, R- Rajpora, SS- Shalimar 1, SR- Shalimar 2, SG- Shalimar 3, TB- Teilbal, NT- Harwan, Z- Zakura; 1, 2, 3- isolate number.

#### Conidial characteristics and shape of phialides

**Fig. 2:**
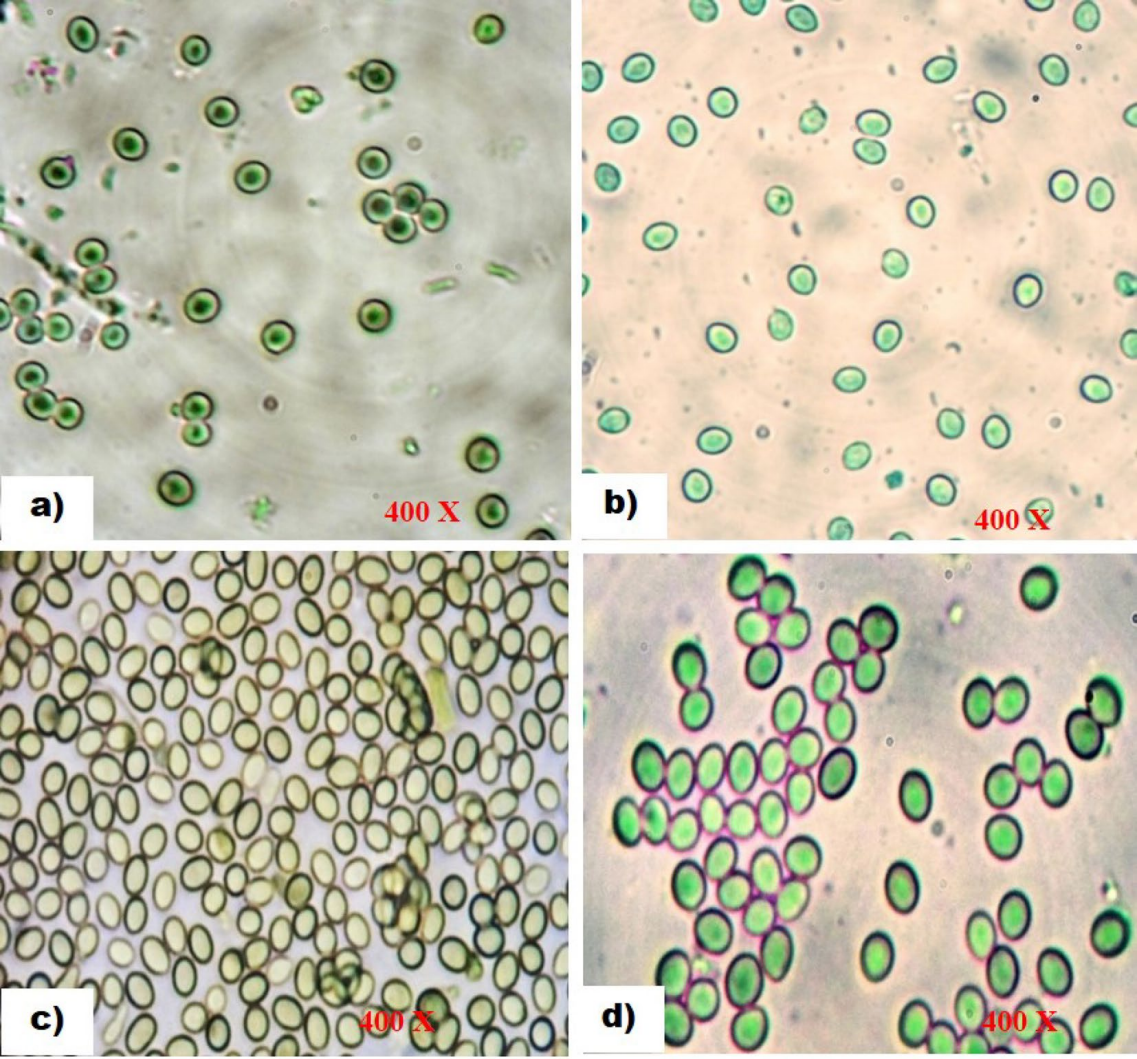
Spore characteristics of *Trichoderma* isolates on PDA after 07 days of incubation at 28 ± 1^o^C; (a) Olive green, globose, smooth conidia; (b) Yellow green, globose to sub-globose, rough conidia; (c) Pale green, globose to sub-cylindrical, smooth conidia; (d) Bright green, sub-globose to obvoid, smooth conidia.

**Fig. 3:**
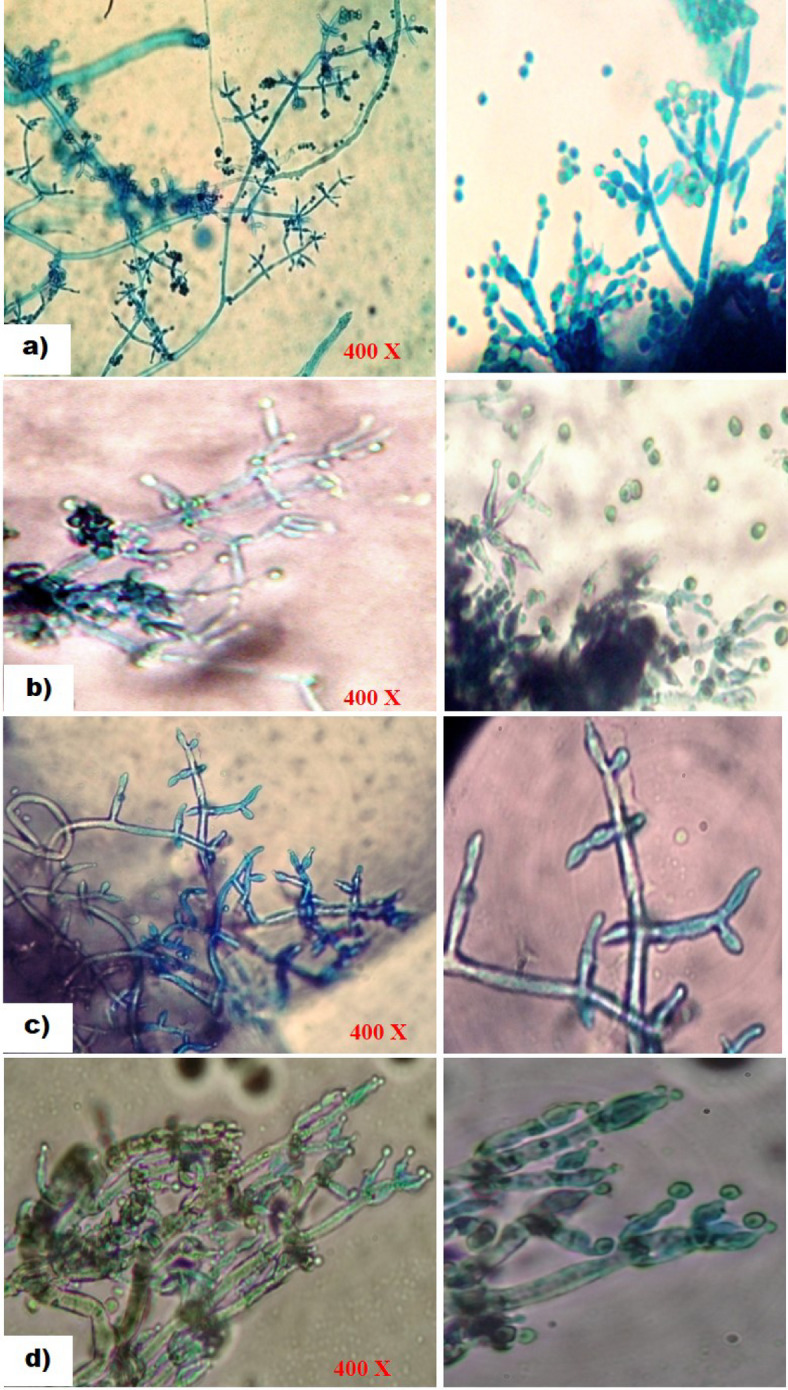
Phialide formation in different *Trichoderma* isolates after 2 days of incubation at 28 ± 1^o^C; (a) Flask shaped phialides; (b) Slender shaped phialides; (c) Elongated shaped Phialides; (d) Swollen shaped phialides.

Different isolates of *Trichoderma* species cultured on PDA medium showed significant variations in their spore characteristics, such as colour, shape, arrangement, and shape of phialides (https://trichoderma.info/). Based on condial and phialide characteristics, *Trichoderma* isolates were categorized into four groups Group I-IV comprised of 10 (Psh1, PTi3, PNi1, PNi3, PR1, PR2, SR, SG, TB2 and TB3), 5 (PNi2, TB1, NT3, Z1 and Z3), 5 (PTi1, PR3, NT1, NT2 and Z2) and 4 (Psh2, Psh3, PTi1 and Z1) isolates. Based on conidial colour, four groups such as Group I with bright green coloured spores (Psh1, PTi3, PNi1, PNi3, PR1, PR2, SR, SG, TB2 and TB3), Group II (yellow-green in isolates PNi2, TB1, NT3, Z1 and Z3), Group III (pale green spores in isolates PTi1, PR3, NT1, NT2 and Z2), and Group IV (olive green spores in isolates Psh2, Psh3, PTi1 and Z1) ) were formed accommodating 10 (41.66%), 5 (20.84%), 5 (20.84%) and 4 (16.66%) isolates, respectively (Table 2). Likewise, the grouping of isolates based on spore shape also resulted in four groups, viz., Group I with sub-globose to obvoid conidia, Group II (globose to sub-globose), Group III (globose to sub-cylindrical), and Group IV (globose-shaped conidia), accommodating 10 (41.66%), 5 (20.84%), 5 (20.84%), and 4 (16.66%) isolates, respectively. Grouping based on spore arrangement, isolates were distributed into two groups namely Group I with smooth-walled conidia accommodated 79.16% of isolates (Psh1, PTi3, PNi1, PNi3, PR1, PR2, SR, SG, TB2, TB3, PTi1, PR3, NT1, NT2, Z2, Psh2, Psh3, PTi1 and Z1), and Group II (rough-walled) comprised 20.84% of isolates (PNi2, TB1, NT3, Z1 and Z3) (Table 2; Fig. 2). Additionally, the isolates were further grouped based on the shape of the phialides into four groups. Group I with flask-shaped phialides, Group II (slender), Group III (elongated), and Group IV (swollen) phialides were formed accommodating 4 (16.60%), 5 (20.80%), 5 (20.80%) and 10 (41.40%) isolates, respectively. Accordingly, grouping of the isolates such as Group I, II, III, and IV was carried out and detailed in Table 2; Fig. 3. Similar observations have been recorded by different researchers all over the world^32–39^ who noted the shape and arrangement of various *Trichoderma* isolates as globose, globose to sub-globose, globose to sub-cylindrical and sub-globose to obvoid having smooth and rough ornamentation. Similarly, it was found that conidial colour also varied widely, including typical green, olive green, grey, brown or even colourless^40^. It has also been reported that the conidia in *Trichoderma* species were olive green or dark green^41^. Furthermore, the grouping based on phialide shape also distributed different isolates into four groups viz., Group I with swollen-shaped phialides, Group II (slender), Group III (elongated), and Group IV (flask) accommodating 10 (41.66%), 5 (20.84%), 5 (20.84%) and 4 (16.66%) isolates, respectively. Other researchers across the globe^16,27,36,39^ have similarly documented that the shape of the phialide of various *Trichoderma* isolates varies from flask to slender, elongated, or swollen, confirming the results obtained from the present study.

**Table 2:** Grouping of *Trichoderma* isolates based on spore colour, shape, arrangement and shape of phialides.

Group	Features	Isolates^$^	Percentage
I	Bright green, smooth walled, sub-globose to obvoid conidia having swollen phialide.	Psh1, PTi3, PNi1, PNi3, PR1, PR2, SR, SG, TB2, TB3	41.66
II	Yellow green, rough walled, globose to sub-globose conidia with slender phialide	PNi2, TB1, NT3, Z1, Z3	20.84
III	Pale green, smooth walled, globose to sub-cylindrical conidia on elongated phialides	PTi1, PR3, NT1, NT2, Z2	20.84
IV	Olive green, smooth walled, globose conidia on flask shaped phialide	Psh2, Psh3, PTi1, Z1	16.66

^**$**^ P- Pulwama, sh- Shadimarg, Ti- Tiken, Ni- Nikas, R- Rajpora, SS- Shalimar 1, SR- Shalimar 2, SG- Shalimar 3, TB- Teilbal, NT- Harwan, Z- Zakura; 1, 2, 3- isolate number.

*Trichoderma* isolates’ conidial size (length x breadth) varied significantly, measuring between 2.50 and 5.00 μm x 2.00–3.80 μm, making it difficult to be grouped solely based on conidial dimensions (Table 3). The maximum mean conidial length of 4.75 μm and breadth of 3.65 μm was observed in isolate SR, followed by isolates PR2 (4.65 × 3.55 μm) and PR3 (4.65 × 3.40 μm) (Table 3). In contrast, the minimum mean conidial length of 2.50 μm and breadth of 2.20 μm was observed in isolate Z1. The highest length and breadth ratio of 1.43 μm was observed in isolate PNi2 and SS, whereas the lowest length and breadth ratio of 1.11 μm was observed in isolate Z1. Previous reports indicated that the conidial size varies from of 2–5 × 2–4 μm in different *Trichoderma* isolates^40^. Similar observations were also recorded by Soesanto et al.^42^who found variability in spore dimensions of four *Trichoderma* isolates. Furthermore, the conidial size was recorded in 30 different *Trichoderma* isolates, and reported that the conidial size and L/W ratio varied significantly in *T. viride* and *T. asperellum*^41^.

**Table 3:** Conidial dimensions of different *Trichoderma* isolates on potato dextrose agar (PDA) medium.

Isolate^$^	Conidial dimensions(µm)^*^		
	Range (LXB)	Mean (LXB)	L/B ratio^**^
Psh1	4.20–4.80 × 3.30–3.50	4.50 × 3.40	1.32
Psh2	2.80–3.20 × 2.40–2.80	3.00 × 2.60	1.15
Psh3	2.50–3.30 × 2.10–2.40	2.90 × 2.20	1.28
PTi1	2.80–3.50 × 2.20–2.50	3.15 × 2.35	1.34
PTi2	3.00-3.50 × 2.50–2.75	3.75 × 2.62	1.24
PTi3	4.30–4.90 × 3.20–3.60	4.60 × 3.40	1.31
PNi1	4.20–4.90 × 3.30–3.70	4.55 × 3.50	1.30
PNi2	3.00-3.60 × 2.20–2.40	3.30 × 2.30	1.43
PNi3	4.20–4.80 × 3.30–3.50	4.50 × 3.40	1.32
PR1	4.30–4.90 × 3.30–3.70	4.60 × 3.50	1.31
PR2	4.50–4.80 × 3.30–3.80	4.65 × 3.55	1.30
PR3	4.40–4.90 × 3.30–3.50	4.65 × 3.40	1.36
SS	3.00-3.60 × 2.20–2.40	3.30 × 2.30	1.43
SR	4.50-5.00 × 3.30–3.80	4.75 × 3.65	1.30
SG	4.25–4.50 × 3.00-3.50	4.37 × 3.25	1.34
TB1	4.50–4.90 × 3.20–3.50	4.70 × 3.35	1.40
TB2	4.20–4.80 × 3.30–3.50	4.50 × 3.40	1.32
TB3	4.30–4.90 × 3.20–3.40	4.60 × 3.30	1.39
NT1	4.50–4.70 × 3.20–3.60	4.60 × 3.40	1.35
NT2	4.20–4.70 × 3.20–3.60	4.45 × 3.40	1.30
NT3	3.30–3.80 × 2.50–2.80	3.55 × 2.65	1.33
Z1	2.20–2.80 × 2.00-2.50	2.50 × 2.25	1.11
Z2	4.40–4.70 × 3.20–3.60	4.55 × 3.40	1.33
Z3	2.90–3.30 × 2.10–2.60	3.10 × 2.35	1.31

^*^Average of 25 microscopic observations (400X); ^**^Length and breadth ratio; ^$^P- Pulwama, sh- Shadimarg, Ti- Tiken, Ni- Nikas, R- Rajpora, SS- Shalimar 1, SR- Shhalimar 2, SG- Shalimar 3, TB- Teilbal, NT- Harwan, Z- Zakura; 1, 2, 3- isolate number.

### Cultural characterization

#### Colony diameter, growth rate and mycelial dry weight

Significant variations were recorded in growth characteristics such as colony diameter, growth rate, and mycelial dry weight of different *Trichoderma* isolates (Table 4). The colony diameter of different isolates ranged from 62.60 to 89.70 mm with the highest mean colony diameter (89.70 mm) recorded in isolate PR2, followed by SR (88.00 mm), PTi1 (84.40 mm), whereas, the least colony diameter (62.60 mm) was recorded in isolate Psh2 (Table 4). Grouping of *Trichoderma* isolates based on cultural characteristics was not possible due to their high level of variation. Similar results were also obtained by different researchers in terms of colony diameter of *Trichoderma* isolates on PDA medium ^30-32,43-45^. The average growth rates varied between 17.63 and 21.31 mm/day, with a maximum growth rate of 21.31 mm/ day recorded in isolates Psh2, TB3, NT2, and Z1 (Table 4). The minimum growth rate of 17.63 mm/ day was observed in Z2 isolate. Variations in growth rates shown by various *Trichoderma* isolates have been well documented by different researchers^45–47^. The average mycelial dry weight of different isolates ranged from 110.36 to 217.67 mg and the highest mycelial dry weight of 217.67 mg was recorded in isolate PR2 followed by SR (197.70 mg), whereas, the lowest mycelial dry weight (110.36 mg) was recorded in isolate PTi3 (Table 4). Significant variations in terms of mycelial dry weight of *Trichoderma* isolates were also reported from different countries^28,47–49^.

**Table 4:** Cultural characteristics of different *Trichoderma* isolates on potato dextrose agar (PDA) medium.

Isolate^$^	Colony diameter^*^ (mm)	Growth rate^***^ (mm/day)	Mycelial dry weight^**^ (mg)
Psh1	67.10 ± 0.454^b^	21.31 ± 9.88	116.17 ± 0.635^c^
Psh2	62.60 ± 0.487^a^	21.25 ± 10.26	110.40 ± 0.583^a^
Psh3	72.80 ± 0.480^g^	21.21 ± 9.10	134.89 ± 0.605^i^
PTi1	84.40 ± 0.285^n^	21.25 ± 11.48	173.47 ± 0.914^s^
PTi2	72.65 ± 0.675^fg^	21.22 ± 8.41	121.51 ± 0.693^e^
PTi3	72.85 ± 0.379^g^	21.27 ± 9.23	110.36 ± 0.694^a^
PNi1	81.80 ± 0.647^l^	21.25 ± 13.68	167.20 ± 0.678^o^
PNi2	75.30 ± 0.325^h^	21.21 ± 10.23	159.45 ± 0.122^m^
PNi3	77.55 ± 0.410^j^	21.25 ± 11.72	168.94 ± 0.901^p^
PR1	82.50 ± 0.176^m^	21.25 ± 14.14	158.39 ± 0.585^m^
PR2	89.70 ± 0.209^p^	21.25 ± 14.43	217.67 ± 0.963^u^
PR3	80.80 ± 0.480^k^	21.30 ± 13.01	145.52 ± 1.150^j^
SS	75.25 ± 0.395^h^	21.28 ± 8.32	151.65 ± 0.255^l^
SR	88.00 ± 0.480^o^	21.25 ± 13.34	197.70 ± 0.478^t^
SG	70.75 ± 0.395^c^	21.27 ± 6.52	120.92 ± 0.478^e^
TB1	72.90 ± 0.454^g^	21.20 ± 9.88	133.56 ± 1.295^h^
TB2	81.10 ± 0.223^k^	21.22 ± 10.77	148.73 ± 0.650^k^
TB3	82.35 ± 0.285^lm^	21.31 ± 14.60	172.19 ± 0.504^r^
NT1	73.15 ± 0.487^g^	21.22 ± 9.24	123.52 ± 0.343^f^
NT2	76.15 ± 0.487^i^	21.31 ± 11.14	165.66 ± 0.982^n^
NT3	82.45 ± 0.410^m^	21.26 ± 14.28	170.31 ± 0.241^q^
Z1	71.40 ± 0.602^d^	21.31 ± 9.79	118.44 ± 0.311^d^
Z2	72.15 ± 0.627^ef^	17.63 ± 8.91	124.88 ± 0.357^g^
Z3	71.85 ± 0.418^de^	21.30 ± 10.97	112.45 ± 0.380^b^
CD (0.05)	Isolate*Time: 0.680		-

^*^Mean of 3 replications at 28 ± 1^**o**^C of incubation; ^**$**^ P- Pulwama, sh- Shadimarg, Ti- Tiken, Ni- Nikas, R- Rajpora, SS- Shalimar 1, SR- Shalimar 2, SG- Shalimar 3, TB- Teilbal, NT- Harwan, Z- Zakura; 1, 2, 3- isolate number; Values superscripted with same alphabet are not significantly different.

Thus, based on morpho-cultural characterization, the 24 isolates of *Trichoderma* were identified up to the species level and grouped into four species. The 4 isolates, namely Psh2, Psh3, PTi2 and Z1, belong to *T. harzianum*, whereas the 6 isolates PNi2, SS, TB1, NT1, NT3 and Z3 were identified as *T. viride* as described previously^17,18,29,35,37,39,40^. Four isolates namely PTi1, PR3, NT2 and Z2 were categorized as *T. koningiopsis* and 10 isolates Psh1, PTi3, PNi1, PNi3, PR1, PR2, SR, SG, TB2 and TB3 were grouped as *T. hamatum* based on the previous descriptions^16,17,39^ (Table 5).

**Table 5:** Identification of *Trichoderma* isolates upto species level based on morpho-cultural characterization.

Isolates^$^ (percentage)	Identified species^*^
Psh2, Psh3, PTi1, Z1 (16.66%)	*T. harzianum*
PNi2, SS, TB1, NT1, NT3, Z3 (25.00%)	*T. viride*
PTi1, PR3, NT2, Z2 (16.66)	*T. koningiopsis*
Psh1, PTi3, PNi1, PNi3, PR1, PR2, SR, SG, TB2, TB3 (41.66%)	*T. hamatum*

^*^T- *Trichoderma*; ^**$**^ P- Pulwama, Sh- Shadimarg, Ti- Tiken, Ni- Nikas, R- Rajpora, SS- Shalimar 1, SR- Shalimar 2, SG- Shalimar 3, TB- Teilbal, NT- Harwan, Z- Zakura; 1, 2, 3- isolate number.

### Molecular characterization of *Trichoderma* species

**Fig. 4:**
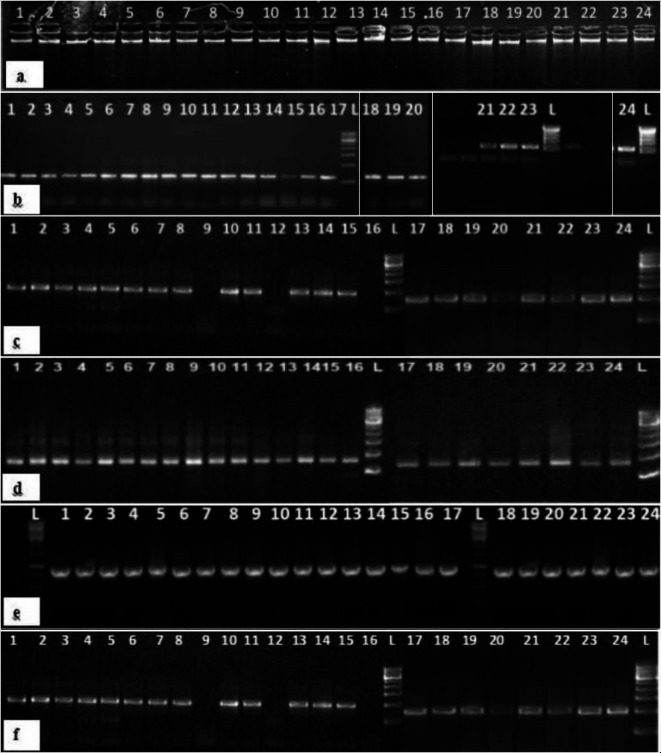
Electrophoretic separation of PCR products of *Trichoderma* isolates (Images showing full length membranes are given in Supplementary Fig. 1) Lane L: DNA Ladder 500 bp; Lanes 1–24: Twenty four *Trichoderma* isolates; a) DNA of 24 *Trichoderma* isolates. b) PCR amplification of ITS region of 24 isolates using ITS1 & ITS4 primers. c) Touchdown PCR amplification of *TEF 1-α*gene using TEF1 & TEF2 primers. d) Internal PCR amplification of *TEF 1-α* gene using TEF3 & TEF4 primers. e) Touchdown PCR amplification of *RPB2* gene using RPB2-5 & RPB2-6 primers. f) Internal PCR amplification of *RPB2* gene using RPB2-5 & RPB2-7 primers.

The DNA of 24 *Trichoderma* isolates was quantified using NanoDrop and then diluted to 40-50ng/µl. High-quality DNA showing a single intact band on a 0.8% agarose gel, was stored at -80℃ in a deep freezer (Eppendorf India Pvt. Ltd.) (Fig. 4a).

### Phylogenetic analysis of multigene sequence data

**Fig. 5:**
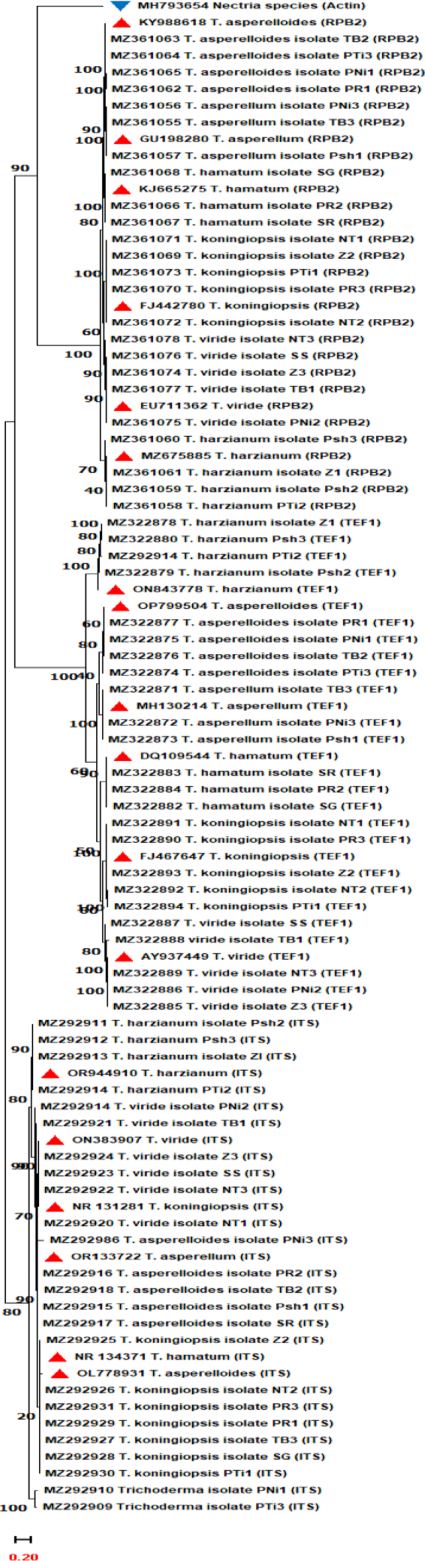
Phylogenetic relationship among *Trichoderma* species based on Internal transcribed spacer (ITS) region, translation elongation factor 1-alpha (*TEF 1-α*) and RNA polymerase B II (*RPB2*) genes. The reference/ out group sequences are highlighted by red/ blue bullets.

The identification of various *Trichoderma* isolates initially based on morpho-cultural characterization was further validated by sequence analysis using molecular markers viz., internal transcribed spacer (ITS) region, translation elongation factor 1-alpha (*TEF 1-α*) and RNA polymerase B II (*RPB2*). The amplification of ITS region with primers ITS1 and ITS4 yielded a product of approximately 600 bp (Fig. 4b). The amplification of *TEF 1-α* gene carried out by touchdown PCR using primer pair 1 and 2 showed an amplicon size of approximately 920 bp. The internal PCR was subsequently performed using primers 3 and 4 amplifying a fragment of approximately 870 bp with the first PCR product serving as a template (Fig. 4c-d). Similarly, touchdown PCR of *RPB2* gene using primer combinations 5 and 6 yielded approximately 1240 bp amplicon size, and the internal PCR showed an amplicon size of about 940 bp using primer pair 5 and 7, respectively (Fig. 4e-f). Accordingly, PCR amplified products of ITS region, and *TEF 1-α* and *RPB2* genes from all the 24 isolates of *Trichoderma* were then custom sequenced (Fig. 4b-f) and results obtained in the form of chromatograms. Based on a concreated data set from ITS region, and *TEF 1-α*, and *RPB2* genes, the 24 isolates of *Trichoderma* were broadly grouped into three major clades (A, B and C), then further sub-divided into sub-clades. Clade A based on ITS region sequences grouped the 24 isolates into five sub-clades viz., Clade I, II, III, IV and V accommodating 8.33, 16.67, 25.00, 20.84 and 29.16% of isolates, respectively (Table 6; Fig. 5). The isolates in clade I were identified as *Trichoderma* species (2 isolates), Clade II as *T. harzianum* (4 isolates), Clade III as *T. viride* (6 isolates), Clade IV as *T. asperelloides* (5 isolates) and Clade V as *T. koningiopsis* (7 isolates) (Table 6; Fig. 5). This study marks a novel attempt to study the phylogeny of the entire *Trichoderma* genus based on sequencing of ITS region, highlighting it as a powerful tool for authentic identification of *Trichoderma* species^50^. However, our findings indicated that the results obtained from the ITS region were not congruent with the morpho-cultural data. This could be possible because few morphological characters with limited variation may lead to overlap and misidentification of the isolates/strains also described by other researchers^51–54^. While the ITS region serves as a valuable identification tool, it is crucial to use identifications based on ITS region with caution, as closely related species such as *T. viride*, *T. harzianum* etc., can share the same ITS sequence, making accurate identification challenging. Thus, relying solely on ITS region is not sufficient for the precise identification and characterization of *Trichoderma* and *Hypocrea* at species level^8,9,54,55^ Therefore, to effectively resolve new species, in addition to ITS region, we employed multiple genes, including *RPB2* and *TEF 1-α*, facilitating a more accurate delineation of *Trichoderma* species from the apple rhizosphere. Notably, over 80 species represented in GenBank are characterized by sequences from at least the ITS region of rDNA, and many are primarily identified using protein-coding genes like *TEF 1-α* and *RPB2*. This clearly underscores the importance of these genes for the precise identification of the *Trichoderma* genus at the species level^10,56–58^.

**Table 6:** Grouping of *Trichoderma* isolates based on phylogenetic analysis of internal transcribed spacer (ITS) region.

Species	Isolates^$^	Percentage
*T.* species	PTi3, PNi1	8.33
*T. harzianum*	Psh2, Psh3, Z1, PTi2	16.67
*T. viride*	PNi2, SS, TB1, NT1, Z3, NT3	25.00
*T. asperelloides*	Psh1, PR2, SR, TB2, PNI3	20.84
*T. koningiopsis*	Z2, SG, PTi1, NT2, TB3, PR1, PR3	29.16

^*^T- *Trichoderma*; ^**$**^ P- Pulwama, sh- Shadimarg, Ti- Tiken, Ni- Nikas, R- Rajpora, SS- Shalimar 1, SR- Shalimar 2, SG- Shalimar 3, TB- Teilbal, NT- Harwan, Z- Zakura; 1, 2, 3- isolate number.

The phylogenetic analysis of sequences based on *TEF 1-α* (clade B) and *RPB2* (clade C) revealed similar results (Fig. 5). Consequently, twenty-four isolates of *Trichoderma* were grouped into six sub-clades viz., Clade I-VI. The isolates in Clade I were identified as *T. koningiopsis*, Clade II as *T. viride*, Clade III as *T. asperellum*, Clade IV as *T. asperelloides*, Clade V as *T. hamatum* and Clade VI as *T. harzianum* accommodating 20.83, 20.83, 12.50, 16.67, 12.50 and 16.67% of isolates, respectively (Table 7; Fig. 5). Other researchers have similarly emphasized the significance of *TEF 1-α* and *RPB2* sequencing in the identification and phylogenetic analysis of *Trichoderma* species^59–63^. Notably, despite the high conservation of the ITS region across the species, results obtained from *TEF 1-α* and *RPB2* genes differed slightly from those obtained from the ITS region. This discrepancy may contribute to misidentifications, and could be attributed to the presence of non-orthologous copies of ITS in these isolates^23,63–66^. Consequently, the variability of *TEF 1-α* and *RPB2* genes facilitates the differentiation among the closely related *Trichoderma* species^11^. Their presence as a single copy in the genomes of all eukaryotes combined with slow rate of divergence making these genes very useful for the higher level of phylogenetic reconstruction^55,63,65–67^.

**Table 7:** Identification of species based on phylogenetic analysis of translation elongation factor 1-alpha (*TEF 1-α*) and RNA polymerase B II (*RPB2*) genes.

Species^*^	Isolates^$^	Percentage
*T. asperelloides*	PTi3, PNi1, PR1, TB2	16.67
*T. asperellum*	Psh1, PNi3, TB3	12.50
*T. hamatum*	PR2,SR, SG	12.50
*T. viride*	PNi2, SS, TB1, NT3, Z3	20.83
*T. koningiopsis*	PTi1, PR3, NT1, NT2, Z2	20.83
*T. harzianum*	Psh2, Psh3, PTi2, Z1	16.67

^*^T- *Trichoderma*; ^**$**^ P- Pulwama, sh- Shadimarg, Ti- Tiken, Ni- Nikas, R- Rajpora, SS- Shalimar 1, SR- Shalimar 2, SG- Shalimar 3, TB- Teilbal, NT- Harwan, Z- Zakura; 1, 2, 3- isolate number.

This study demonstrated that the morpho-cultural or molecular characterization independently did not produce congruent results. Consequently, definitive identification of various *Trichoderma* species was achieved through an integrated approach that combined morpho-cultural as well as molecular characterization based on ITS region, and *TEF 1-α* and *RPB2* genes. This analysis resulted in the categorization of 24 isolates into six distinct groups viz., Group I (*T. koningiopsis*), Group II (*T. viride*), Group III (*T. asperellum*), Group IV (*T. asperelloides*), Group V (*T. hamatum*) and Group VI (*T. harzianum*) accommodating 5 (PTi1, PR3, NT1, NT2, Z2), 5 (PNi2, SS, TB1, NT3, Z3), 3 (Psh1, PNi3, TB3), 4 (PTi3, PNi1, PR1, TB2), 3 (PR2, SR, SG) and 4 (Psh2, Psh3, PTi2, Z1) isolates, respectively. These findings indicated the presence of six species of *Trichoderma* in apple rhizosphere in two districts of Srinagar and Pulwama (Table 8). The consensus sequences based on ITS region, *TEF 1-α* and *RPB2* genes of *Trichoderma* species were used in the nucleotide blast program of the NCBI database for the identification of each isolate and submitted to the GenBank NCBI, and Accession numbers obtained from MZ292909 to MZ292931 and MZ292986, MZ322871 to MZ322894 and MZ361055 to MZ361078, respectively (Table 8). To our knowledge, this study reports for the first time the occurrence of *T. koningiopsis*, *T. viride*, *T. asperellum*,* T. asperelloides* and *T. hamatum* in apple rhizosphere from India, although, their presence in the rhizosphere of other crops is well documented.

**Table 8:** Final confirmation of *Trichoderma* isolates based on morpho-cultural and molecular characterization.

Isolates^$^ No.	Accession Number			Identified species^*^	Percentage
	ITS	*TEF 1-α*	*RPB2*		
PTi1	MZ292930	MZ322894	MZ361073	*T. koningiopsis*	20.83
PR3	MZ292931	MZ322890	MZ361070		
NT1	MZ292920	MZ322891	MZ361071		
NT2	MZ292926	MZ322892	MZ361072		
Z2	MZ292925	MZ322893	MZ361069		
PNi2	MZ292914	MZ322886	MZ361075	*T. viride*	20.83
SS	MZ292923	MZ322887	MZ361076		
TB1	MZ292921	MZ322888	MZ361077		
NT3	MZ292922	MZ322889	MZ361078		
Z3	MZ292924	MZ322885	MZ361074		
Psh1	MZ292915	MZ322873	MZ361057	*T. asperellum*	12.50
PNi3	MZ292986	MZ322872	MZ361056		
TB3	MZ292927	MZ322871	MZ361055		
PTi3	MZ292909	MZ322874	MZ361064	*T. asperelloides*	16.67
PNi1	MZ292910	MZ322875	MZ361065		
PR1	MZ292929	MZ322877	MZ361062		
TB2	MZ292918	MZ322876	MZ361063		
PR2	MZ292916	MZ322884	MZ361066	*T. hamatum*	12.50
SR	MZ292917	MZ322883	MZ361067		
SG	MZ292928	MZ322882	MZ361068		
Psh2	MZ292911	MZ322879	MZ361059	*T. harzianum*	16.67
Psh3	MZ292912	MZ322880	MZ361060		
PTi2	MZ292914	MZ322881	MZ361058		
Z1	MZ292913	MZ322878	MZ361061		

^*^T- *Trichoderma*; ^**$**^ P- Pulwama, sh- Shadimarg, Ti- Tiken, Ni- Nikas, R- Rajpora, SS- Shalimar 1, SR- Shalimar 2, SG- Shalimar 3, TB- Teilbal, NT- Harwan, Z- Zakura; 1, 2, 3- isolate number.

## Materials and methods

### Collection, isolation, purification, and maintenance of fungal isolates

Soil samples from apple rhizosphere were collected from various apple-grown areas viz., Nikas, Tiken, Rajpora, and Shadimarg in district Pulwama and Shalimar, New Theed, Harwan, and Zakura in district Srinagar of Kashmir valley (Table 9). Three soil samples from each location were collected in triplicates to a depth of 30 cm. The samples from each site were thoroughly mixed and foreign materials like roots, stones, pebbles, and gravel were removed. The bulk was reduced to 0.5–1.0 kg by quartering technique, where the thoroughly mixed samples were divided into four equal parts. The two diagonally opposite segments were discarded and the remaining two segments were recombined and remixed. This procedure was repeated until the desired sample size was achieved. The final samples were collected in paper bags, appropriately labeled, and used for the isolation of *Trichoderma* species. *Trichoderma* strains were isolated using serial dilution plate technique^68^ on *Trichoderma* specific medium (TSM)^26,69–72^ and incubated at 28 ± 1^o^C for one week until sporulation was observed. The different isolates were purified on a water agar medium using a single spore technique^73^. Twenty-four isolates belonging to different species of *Trichoderma* were obtained from two districts, namely Pulwama and Srinagar of Kashmir valley, and coded as per location, etc. (Table 9). The 24 *Trichoderma* cultures were maintained by sub-culturing on potato dextrose agar (PDA) medium, incubated at 28 ± 1^o^C, and stored at -80^o^C for further studies.

**Table 9:** *Trichoderma* isolates obtained from apple rhizosphere along with their code.

District	Location	Isolate code
Pulwama	Shadimarg	Psh1
		Psh2
		Psh3
	Tiken	PTi1
		PTi2
		PTi3
	Nikas	PNi1
		PNi2
		PNi3
	Rajpora	PR1
		PR2
		PR3
Srinagar	Shalimar	SS
		SR
		SG
	Teilbal	TB1
		TB2
		TB3
	Harwan	NT1
		NT2
		NT3
	Zakura	Z1
		Z2
		Z3
**Total No. of isolates**		**24**

### Morpho-cultural characterization

Morphological and cultural characteristics of each isolate were observed on the PDA medium (HiMedia Lab. Pvt. Ltd., Mumbai, India). A 5-mm mycelial disc from each isolate from an actively growing region of a 7-day-old culture was aseptically punched and transferred to Petri plates containing PDA medium, followed by incubation at 28 ± 1 °C. After 5 days of incubation, colony characteristics such as colony texture, margins, colour, and shape were recorded. Conidial morphology was examined by preparing spore suspension from the seven-day-old culture of each isolate and observed under a microscope model CX31 (Olympus, Tokyo, Japan) to record shape, colour, septation, and size (length and width) for at least 25 conidia per isolate. Phialide shape was also assessed after 2 days, and the presence or absence of chlamydospores after 10 days of incubation at 28 ± 1ºC. Cultural characteristics such as colony diameter was recorded for 3 days and growth rate was calculated up to 4 days in all the isolates of *Trichoderma*. The mycelial dry weight of different isolates was calculated by drying the filter papers containing mycelium in a Thermotech hot air oven (MAC-230, Punjab Biotechnology, Chandigarh, India) at 60ºC for an hour for 3 consecutive days till a constant weight was attained. Three replications were maintained for each isolate for all the experiments. The data collected was analyzed by appropriate statistical method^74^.

### Molecular characterization of various *Trichoderma* isolates

#### Genomic DNA extraction

Fungal cultures of all the isolates of *Trichoderma* spp. were separately cultured on potato dextrose broth (PDB) medium in 150 ml Erlenmayer flasks. After sterilization of PDB medium in 250 ml conical flasks at 15 lb psi in an autoclave (MAC-80 L, Punjab Biotechnology, Chandigarh, India) for 20–25 min., flasks were inoculated (at room temperature) with 5 mm mycelial discs of each *Trichoderma* isolate and incubated at 28 ± 1 °C for 7 days. Mycelium was filtered through double-layered sterilized filter paper, dried between two layers of filter paper in a laminar airflow cabinet (PCR 2.5, Macro Scientific Works, New Delhi, India), and stored separately at − 80 °C in a deep freezer (U 410, Eppendorf Hamburg · Germany) for further use. The total genomic DNA of each isolate was extracted using the CTAB (Cetyl trimethyl ammonium bromide) method^75^ with slight modifications and diluted to a final concentration of 25 ng/µl.

#### Quantification and quality check of DNA

The quantity of DNA was checked by agarose gel electrophoresis. In this, 0.8 g of agarose was dissolved in 100 ml of 0.5X Tris-acetate EDTA (TAE) (SRL Pvt. Ltd., Mumbai, India) electrophoresis buffer. The mixture was heated until the agarose was dissolved completely i.e. when the solution became transparent and clear. It was cooled down to 60 °C with constant stirring. Ethidium bromide was added to a final concentration of 0.5 µg/ml of buffer. Then the agarose solution was poured into an already prepared gel mould with combs and was left for 20–30 min for solidification. DNA samples for loading were prepared by adding 2 µl loading dye (6X) (0.25% w/v bromophenol blue and 50% glycerol in sterile water) (Thermo Fisher Scientific Pvt. Ltd., India) to 8 µl DNA so that the final concentration of loading dye was 1X. The DNA samples were loaded into wells with the help of a micropipette. Along with the DNA samples, a marker of known concentration was also loaded. The gel was run for about 1–2 h at 80 volts and visualized under a UV transilluminator using a photo gel documentation system (Alfa Imager EC, Protein Simple, USA), and a DNA sample was photographed. The intensity of fluorescence of each sample was compared with that of a standard marker and then the DNA concentration of each sample was ascertained. The quantity of DNA samples was also checked by Nano-drop (Bio-spectrometer, Eppendorf India Pvt. Ltd.). The quality was checked whether DNA formed a single high molecular weight band (good quality) or a smear (degraded/poor quality) in gel electrophoresis. The DNA of all the samples was diluted to 25 ng/µl by adding double-distilled sterile water, stored at − 80 °C in a deep freezer (U 410, Eppendorf Hamburg · Germany), and used for PCR amplification.

### Polymerase chain reaction (PCR) using internal transcribed spacer (ITS) region, translation elongation factor 1-alpha (*TEF 1-α*), and RNA polymerase B subunit II (*RPB2*) gene-specific markers

Polymerase chain reaction (PCR) using ITS region, and *TEF 1-α*, and *RPB2* gene-specific markers was performed in 0.2 ml PCR tubes in a T-Gradient thermal cycler (Whatman Biometra, T-Gradient, Goettingen, Germany) using 40–50 ng of genomic DNA of each isolate in a final volume of 50µl reaction mixture comprised of 31.6µl distilled sterilized PCR-water, 5.0µl (1.0X) PCR buffer, 3µl (1.5 mM) MgCl_2)_, 4µl (0.2 mM) DNTPs, 2.0µl of each forward and reverse primers (0.4 pmol) and 0.4µl *Taq* polymerase (1.0 unit) provided by Thermo Fisher Scientific Pvt. Ltd., India. PCR amplification of ITS region was performed in a thermal cycler programmed for initial denaturation at 95℃ for 5 min. followed by 35 cycles of denaturation at 94℃ for 1 min., primer annealing for 2 min. at 56℃, primer extension for 3 min. at 72℃ with a final extension of 10 min^52^. The *TEF 1-α* was amplified using touch-down PCR (primer pair *TEF* 1 and 2) programmed with initial denaturation at 94℃ for 4 min. followed by 4 cycles at 94℃ for 1 min. and 90 sec. each at 70 and 68℃ followed by 26 cycles with annealing temperature decreasing by 0.5℃ per cycle from 68 − 55℃; 12 cycles with annealing at 55℃ and a final extension of 7 min. at 68℃. The internal PCR was carried out using primer pair TEF-3 and 4 programmed at 94℃ for 4 min. followed by 35 cycles at 94℃ for 30 sec., annealing at 50℃ for 15 sec., extension at 68℃ for 1 min. and 7 min., respectively^62^. Similarly, touch-down PCR was performed for *RPB2* amplification using primer pair *RPB2* 5 and 6 programmed at 94℃ for 4 min. followed by 5 cycles at 94℃ for 45 sec., 60℃ for 45 sec. and 68℃ for 2 min. and 5 cycles with annealing temperature decreasing by 1.0℃ per cycle from 58 − 54℃ followed by 30 cycles of annealing at 54℃ and a final extension of 10 min at 68℃. The internal PCR was performed with primer pair *RPB2* 5 and 7 programmed at 94℃ for 4 min. followed by 35 cycles of denaturation at 94℃ for 30 sec., annealing at 50℃ for 15 sec., extension at 68℃ for 1 min. and a final extensioof 7 min. at 68℃ 622. A set of ITS region, and TEF1-α, and *RPB2* gene-specific primers^76-79^ were used for molecular characterization of 24 isolates of *Trichoderma* (Table 10). The ITS region of rDNA was amplified using ITS1 (5’TCCGTAGGTGAACCTGCG3’) and ITS4 (5’TCCTCCGCTTATTTGATATGC3’) primers^76^. *TEF 1-α*^78^ and *RPB2* ^79^ genes were amplified according to already given procedure^79^. A fragment of the 5’ end of *TEF 1-α* gene containing three introns was amplified using a primer combination of 1 (5’CAAAATGGGTAGGAGGASAAGAC3’) and 2 (CAGTACCGGCRGCRATRATSAG3’) following touch-down PCR. This amplified product was re-amplification by an internal PCR using a primer combination of 3 (5’AGGACCAAGACTCACATCAACG3’) and 4 (5’AGTACCAGTGATCATGTTCTTG’3). A fragment of subunit 2 of *RPB2* gene was amplified using primer pair of 5 (5’TGGGGWGAYCARAARAAGG’3) and 6 (5’CATRATGACSGAATCTTCCTGGT’3) following a touch-down PCR followed by an internal PCR using a primer pair of 5 (5’TGGGGWGAYCARAARAAGG’3) and 7 (5’GGTTGTGATCRGGRAARGGAATG’3).

**Table 10:** *TEF 1-α*, *RPB2* and ITS primers, their sequences and annealing temperatures for PCR analysis of different isolates of *Trichoderma* species.

S. No.	Primers	Sequences	Annealing Temperature (ºC)
1	EF-1	5’CAAAATGGGTAAGGAGGASAAGAC’3	68 − 55
2	EF-2	5’CAGTACCGGCRGCRATRATSAG’3	68 − 55
3	EF-3	5’AGGACAAGACTCACATCAACG’3	50
4	EF-4	5’AGTACCAGTGATCATGTTCTTG’3	50
5	*RPB2*-5	5’TGGGGWGAYCARAARAAGG’3	58 − 54
6	*RPB2*-6	5’CATRATGACSGAATCTTCCTGGT’3	58 − 54
7	*RPB2*-7	5’GGTTGTGATCRGGRAARGGAATG’3	50
8	ITS1	5’TCCGTAGGTGAACCTGCG3’	56 56
9	ITS4	5’TCCTCCGCTTATTTGATATGC3’	

### DNA sequencing and phylogenetic analysis

After PCR amplification using ITS, *TEF 1-α*, and *RPB2* primers in a 50-µl reaction mixture, 5 µl of PCR amplified product of different isolates was electrophoresed to ensure successful amplification and the remaining 45 µl PCR amplified products of 24 isolates of *Trichoderma* spp. were sent for custom sequencing to Genei Labs, Bangalore, India. The sequences for each forward and reverse primer were retrieved from the chromatograms received. The sequence alignment was carried out for both forward and reverse sequences in BioEdit version 7.0 ^80^ to obtain consensus sequences for each isolate. Similarly, all the 24 sequences for ITS region, and TEF1-α and *RPB2* genes were obtained and analyzed through the BLASTn program of National Centre for Biotechnological Information (NCBI) (http://www.ncbi.nlm.nih.gov/BLAST) to compare the present sequences with the available database of NCBI (http://www.nlm.nih.gov/nuccore). The consensus sequences of ITS, *TEF 1-α*, and *RPB2* for each isolate obtained were compared with three sequences of each of *T. harzianum*,* T. viride*,* T. koningiopsis*,* T. hamatum*,* T. asperellum*,* T. asperelloides* along with an outgroup sequence retrieved from NCBI database using molecular evolutionary genetic analysis (MEGA) software version 11 ^81^. An optimal tree was generated using the Kimura-2-parameter (K2) substitution model and different taxa were clustered together in a bootstrap test with 1000 replicates^82^. The phylogenetic analysis confirmed the clustering of isolates into distinct clades using the best-fit model. The twenty-four sequences of each gene were submitted to GenBank, NCBI for the allotment of Accession numbers.

## Conclusion

In conclusion, this study has unveiled a remarkable diversity of *Trichoderma* species within the apple rhizosphere of Jammu and Kashmir, India. It highlights the critical importance of integrating morpho-cultural and molecular methodologies for the precise characterization of these fungi. Our findings demonstrate that relying on a single approach is insufficient for identifying the various *Trichoderma* isolates at the species level. Thus, by employing a multifaceted strategy that combines morpho-cultural characterization with advanced molecular techniques—including the sequencing of the ITS region, *TEF 1-α*, and *RPB2* genes—we can achieve a clearer delineation of these species. This approach led to the successful identification of *T. koningiopsis*, *T. viride*, *T. asperellum*,* T. asperelloides*,* T. hamatum* and *T. harzianum*, all of which were isolated from apple rhizosphere in Jammu and Kashmir, India. This research not only deepens our understanding of *Trichoderma* biodiversity but also opens avenues for future investigations into the ecological roles of these fungi and their potential as biocontrol agents in sustainable agricultural practices. To our knowledge, this is the first report of *T. koningiopsis*, *T. viride*, *T. asperellum*,* T. asperelloides* and *T. hamatum* from apple rhizosphere.

## Electronic supplementary material

Below is the link to the electronic supplementary material.

Supplementary Material 1

## Data Availability

The sequence data of this paper has been deposited in GenBank, NCBI and accession numbers (MZ292909 to MZ292931 and MZ292986, MZ322871 to MZ322894 and MZ361055 to MZ361078) are given in the paper.
