# Supplementary material for: Morpho-cultural and molecular characterization of trichoderma species from the northwestern himalayan apple rhizosphere of India
Source: Sci Rep. 2025 Jul 20;15:26320. doi: 10.1038/s41598-025-12086-4 (PMC12277453; doi:10.1038/s41598-025-12086-4)
Supplement: Supplementary file 1 — Supplementary Material 1 [file 41598_2025_12086_MOESM1_ESM.docx]

**Supplementary Fig. 1 Original gels without crop or modification showing DNA check and PCR amplification of ITS region, TEF 1-α, and RPB2 genes**

**DNA CHECK**


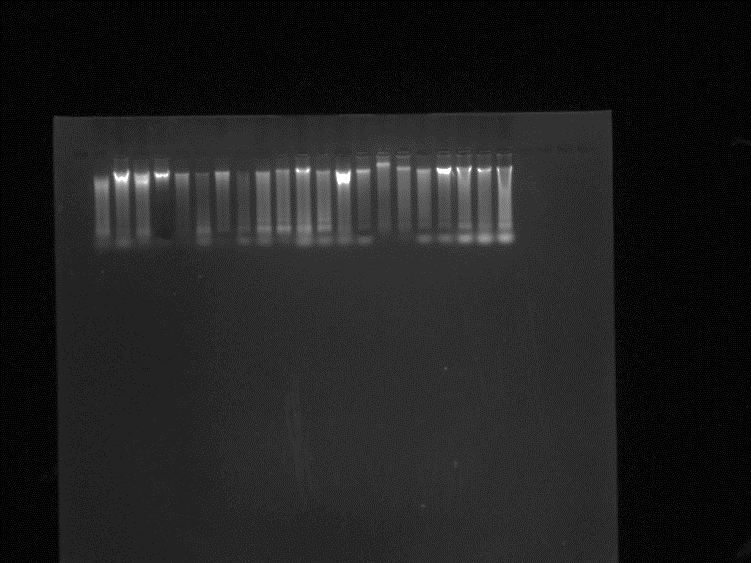


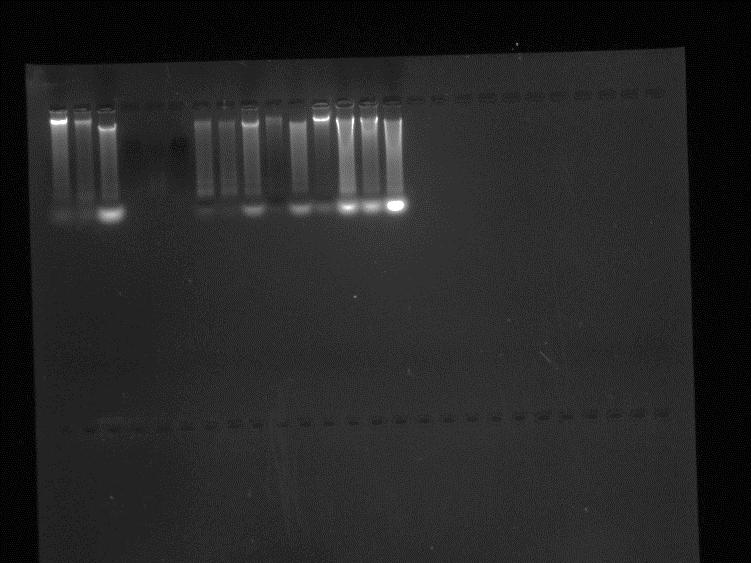


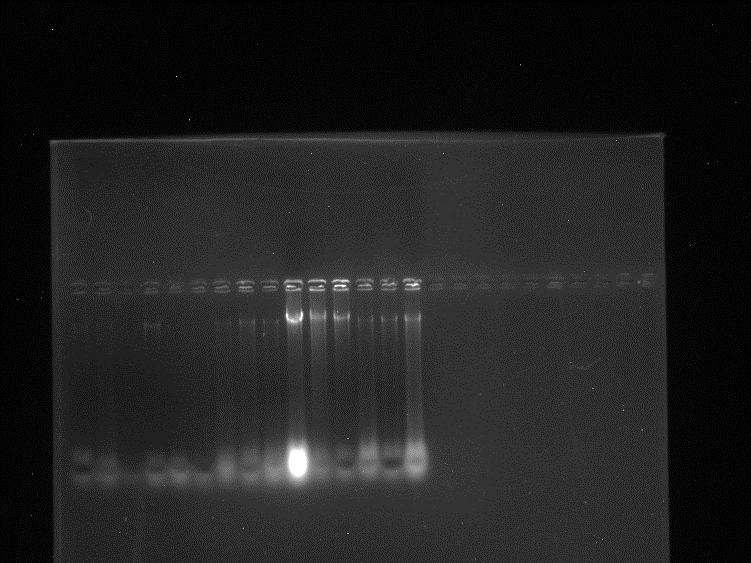


**ITS**

**
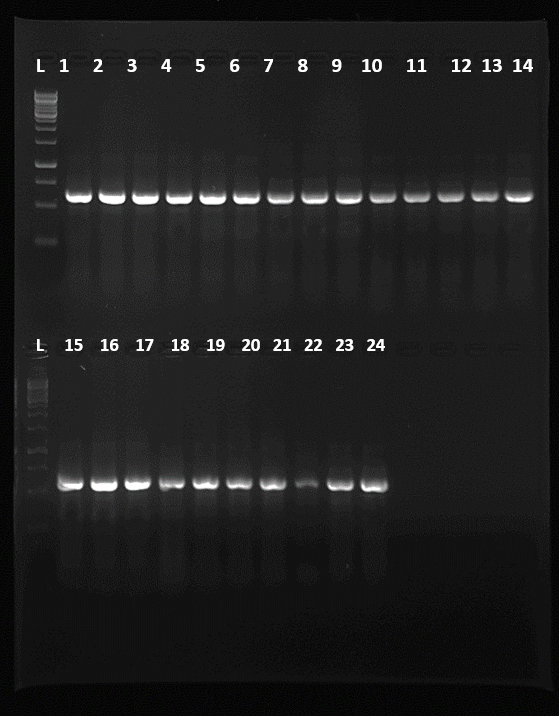
**
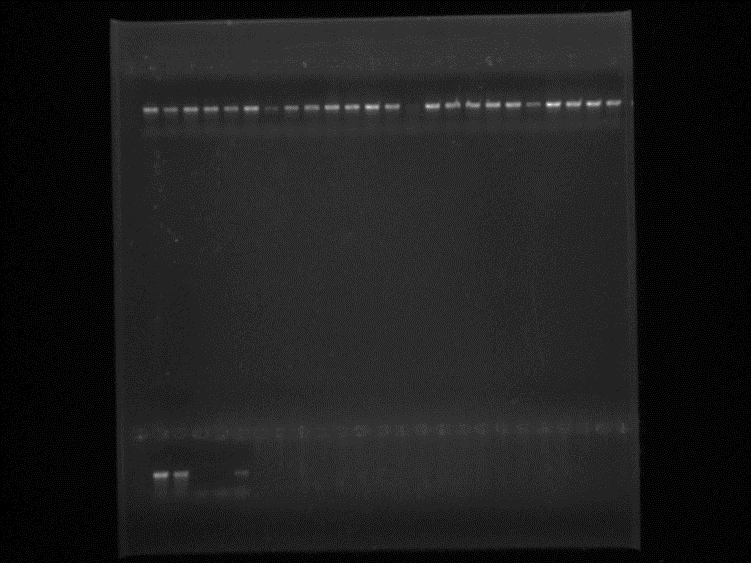


A

Ladder: 1kb


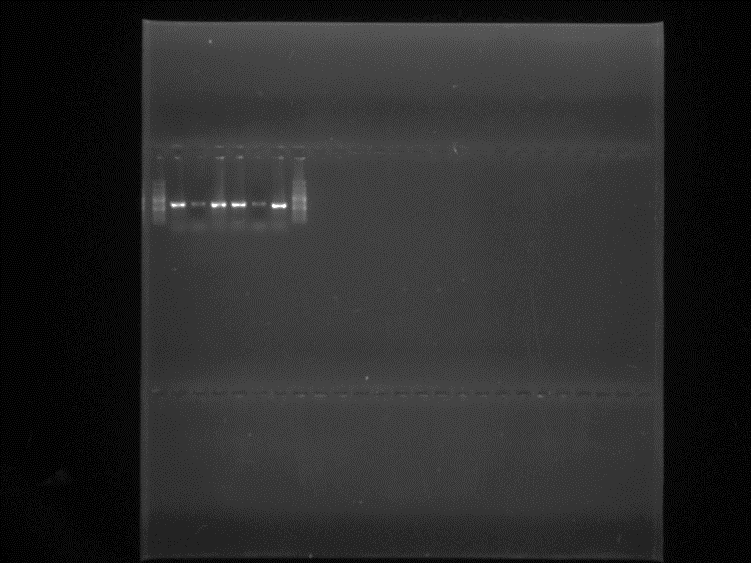

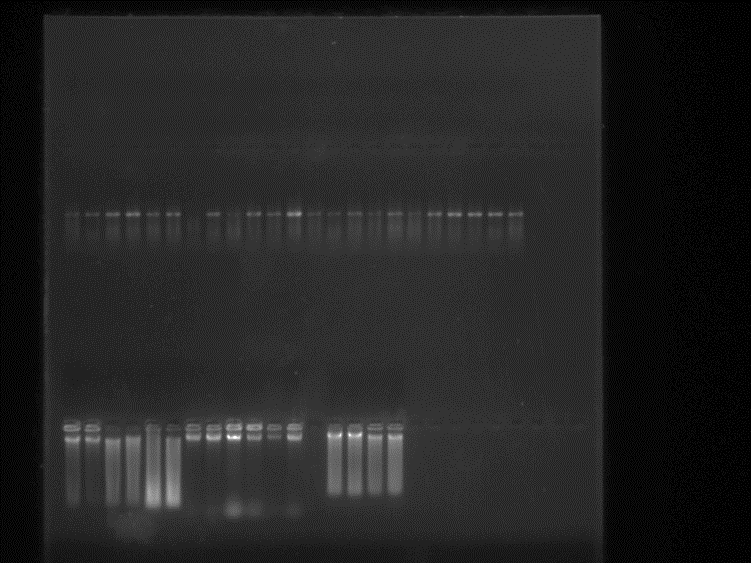


**TEF 1-alpha (TEF 1-α)-These 2 gels are the recent ones**

**
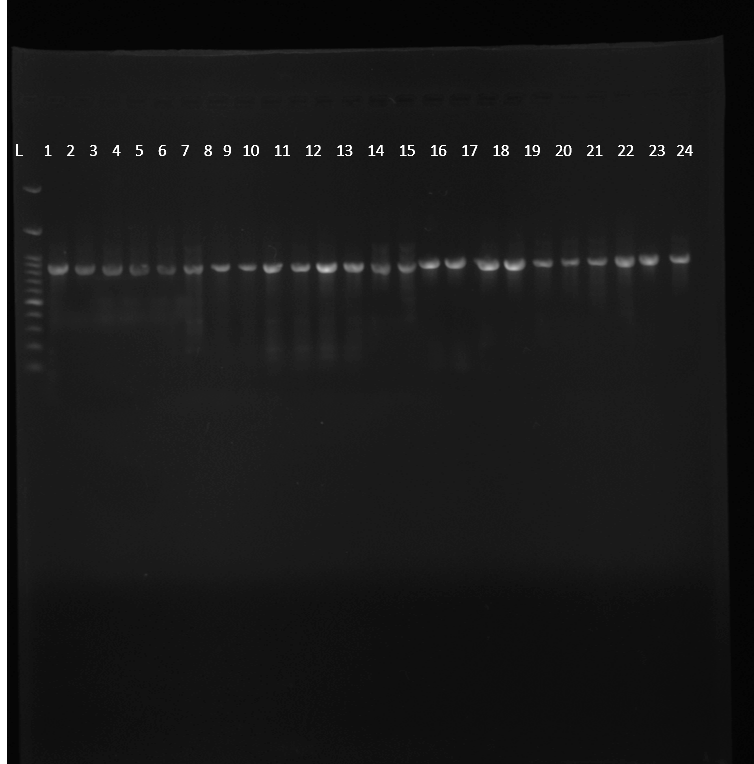

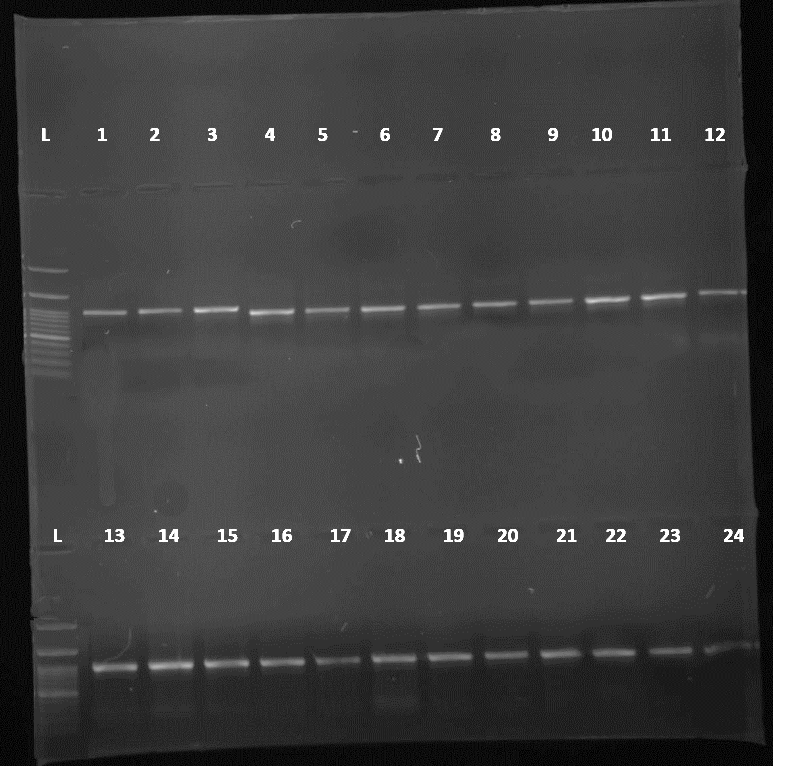
**

**C**

**B**

**TEF 1-α 1 and 2; Ladder: 100bp TEF 1-α 3 and 4; Ladder: 100bp**


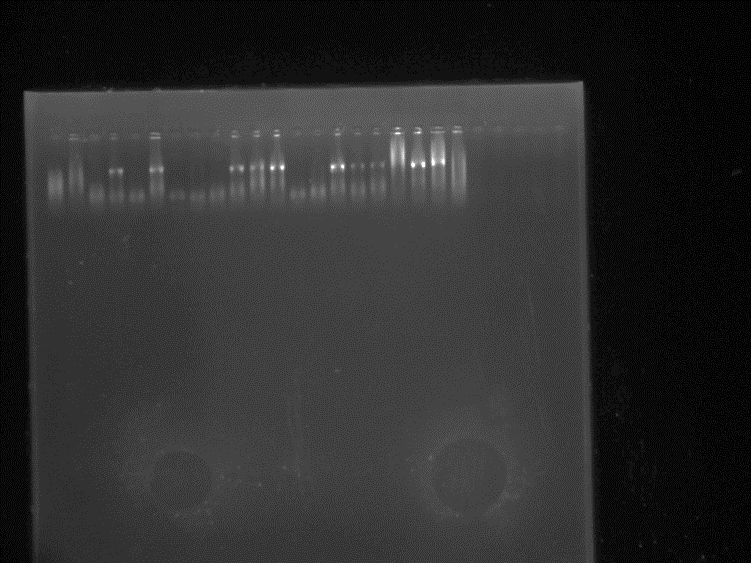

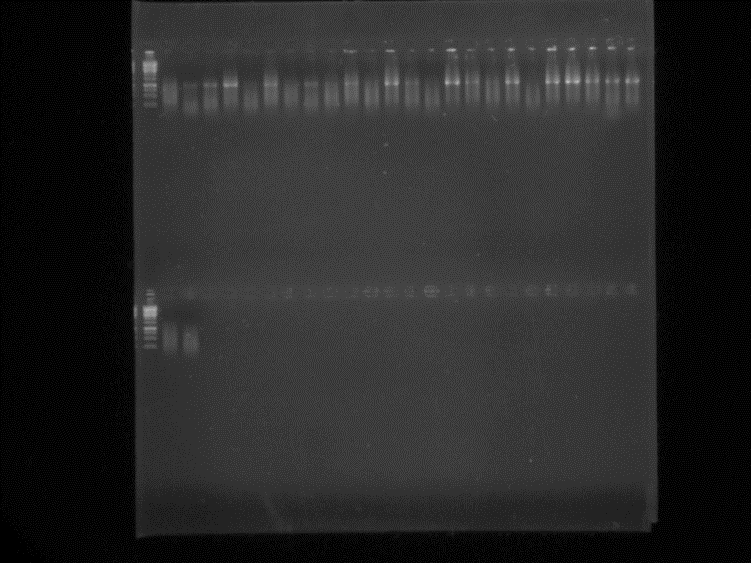


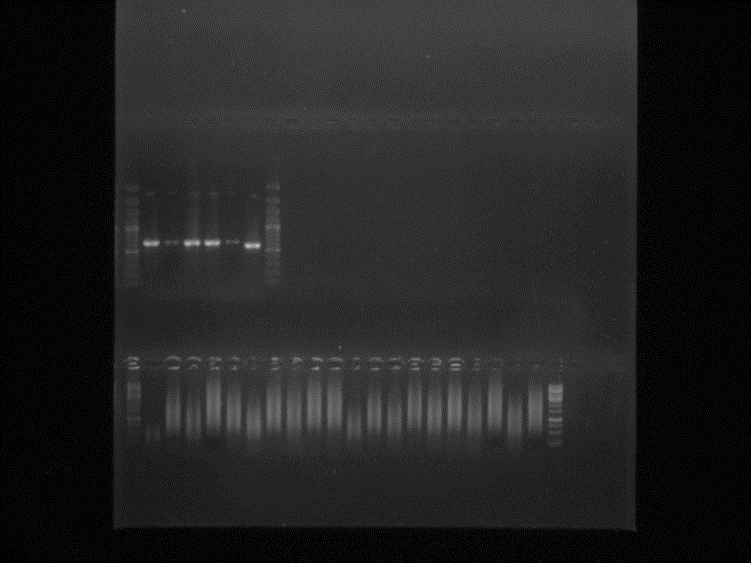

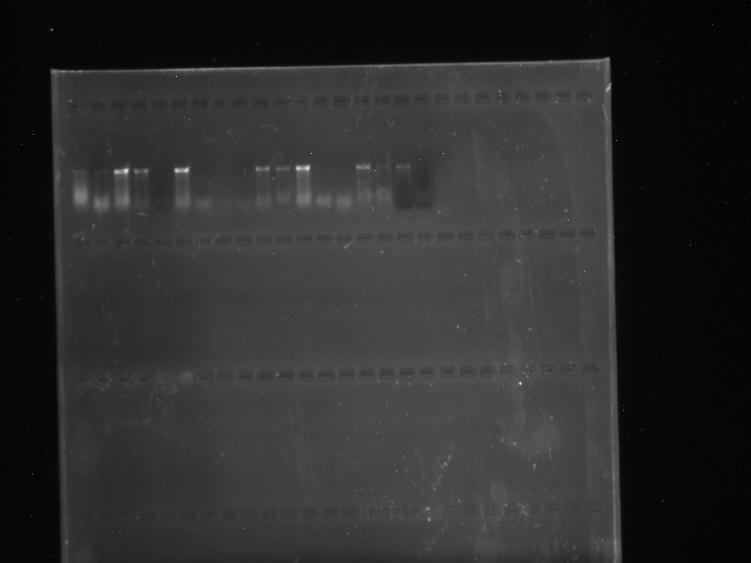


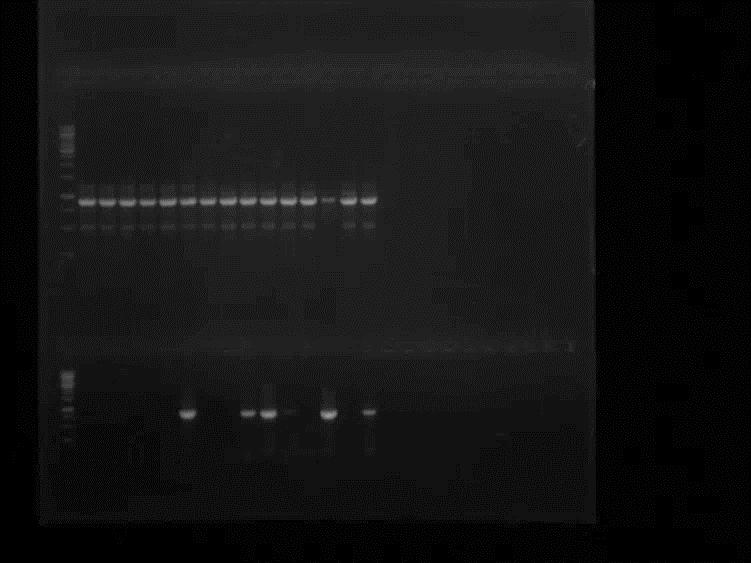

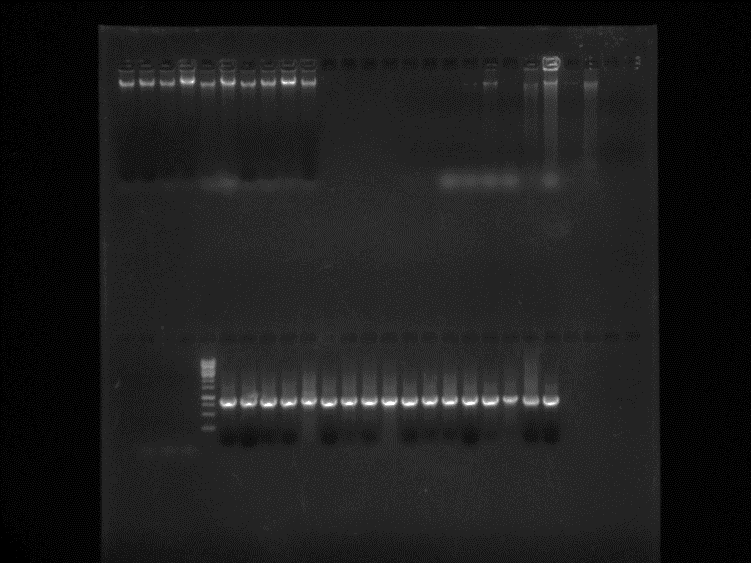


**RPB2**

**
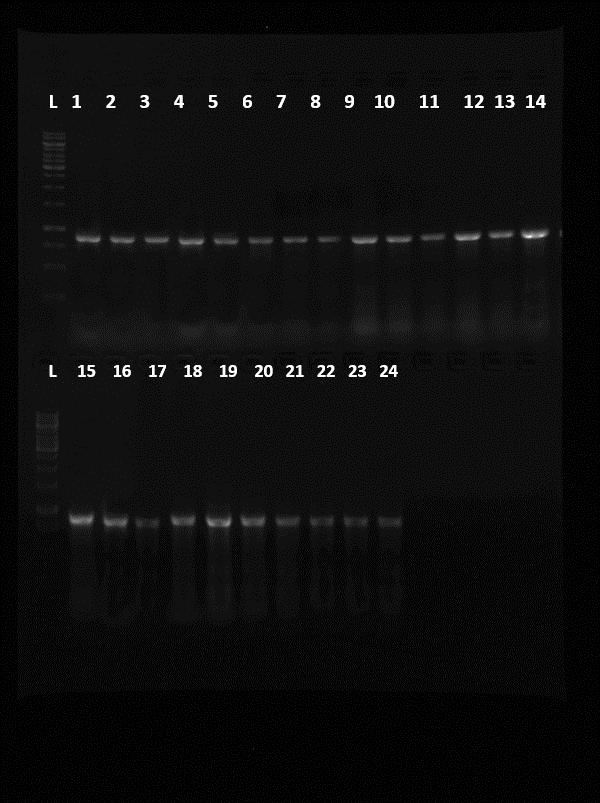

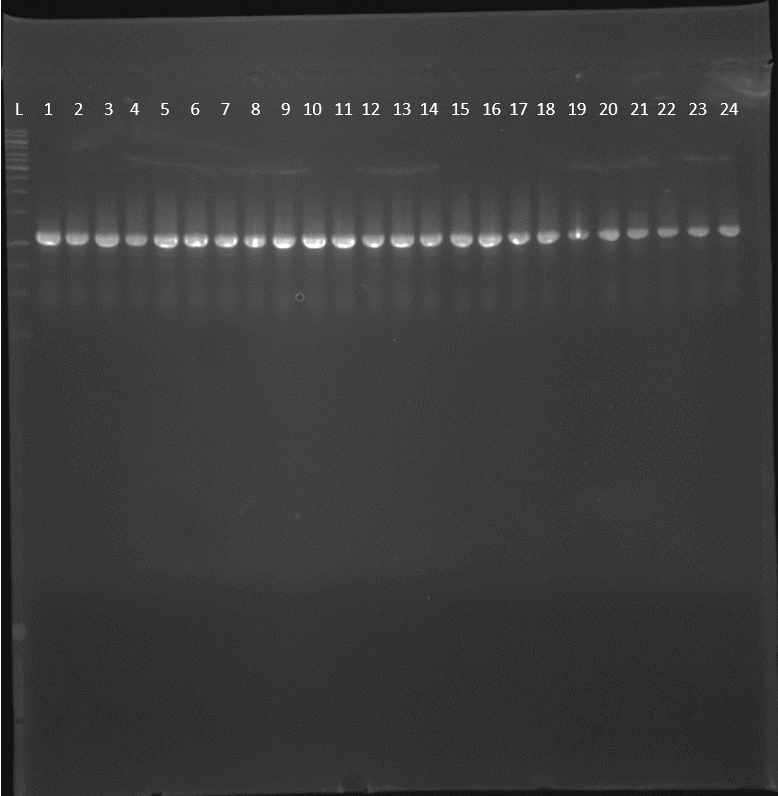
**

**E**

**D**

**RPB2 5 and 7; Ladder 1kb**

**
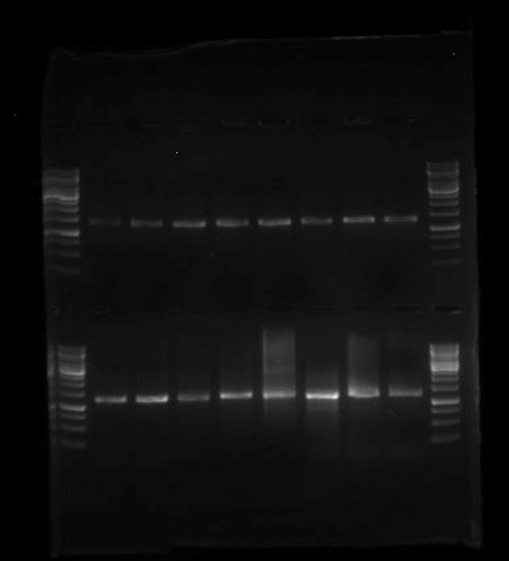
**
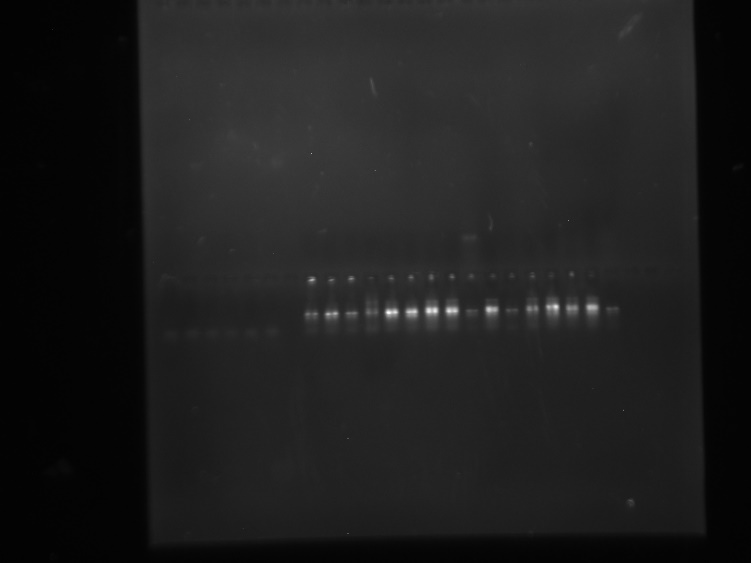


**F**

**RPB2 5 and 6; Ladder- 1kb**


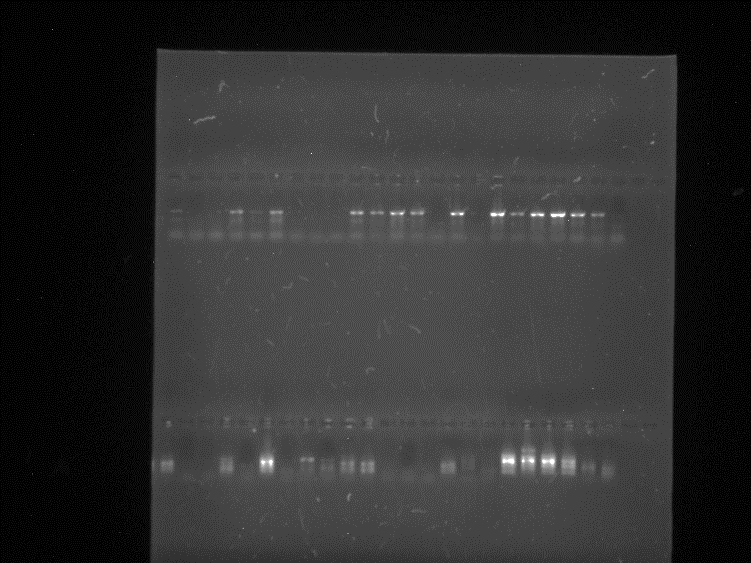

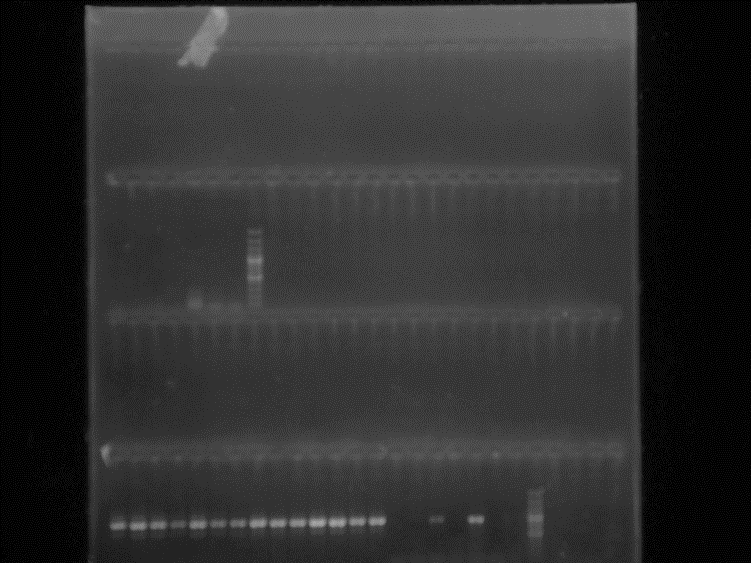


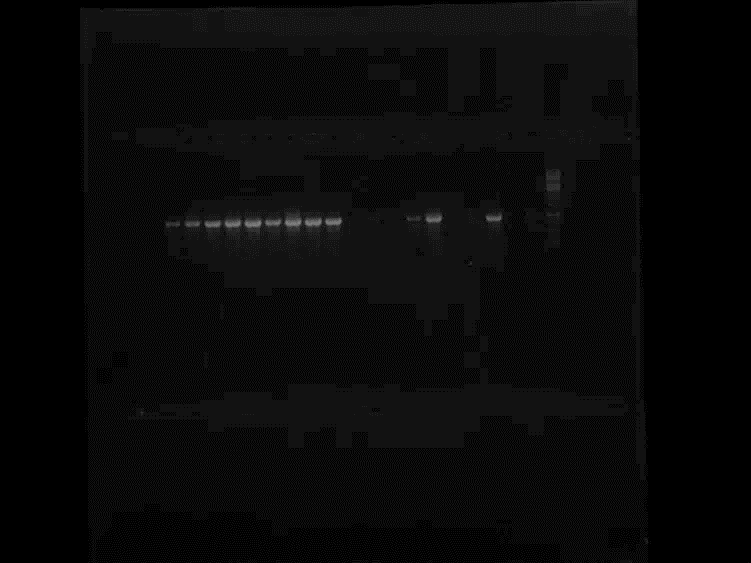

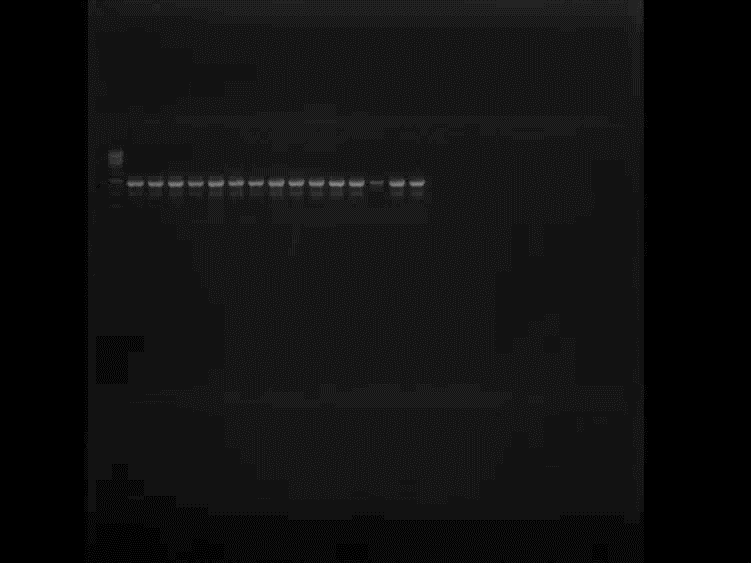


RPB2 5 and 7
